# Electrophysiology Reveals the Neural Dynamics of Naturalistic Auditory Language Processing: Event-Related Potentials Reflect Continuous Model Updates

**DOI:** 10.1523/ENEURO.0311-16.2017

**Published:** 2017-12-08

**Authors:** Phillip M. Alday, Matthias Schlesewsky, Ina Bornkessel-Schlesewsky

**Affiliations:** 1 Department of the Psychology of Language, Max-Planck-Institute for Psycholinguistics, Nijmegen 6500AH, The Netherlands; 2 Cognitive Neuroscience Laboratory, School of Psychology, Social Work and Social Policy, University of South Australia, Adelaide SA 5001, Australia

**Keywords:** ecological validity, mixed-effects models, naturalistic stimuli, predictive coding

## Abstract

The recent trend away from ANOVA-based analyses places experimental investigations into the neurobiology of cognition in more naturalistic and ecologically valid designs within reach. Using mixed-effects models for epoch-based regression, we demonstrate the feasibility of examining event-related potentials (ERPs), and in particular the N400, to study the neural dynamics of human auditory language processing in a naturalistic setting. Despite the large variability between trials during naturalistic stimulation, we replicated previous findings from the literature: the effects of frequency, animacy, and word order and find previously unexplored interaction effects. This suggests a new perspective on ERPs, namely, as a continuous modulation reflecting continuous stimulation instead of a series of discrete and essentially sequential processes locked to discrete events.

## Significance Statement

Laboratory experiments on language often lack ecological validity. In addition to the intrusive laboratory equipment, the language used is often highly constrained in an attempt to control possible confounds. More recent research with naturalistic stimuli has been largely confined to fMRI, where the low temporal resolution helps to smooth over the uneven finer structure of natural language use. Here, we demonstrate the feasibility of using naturalistic stimuli with temporally sensitive methods such as electroencephalography (EEG) and magnetoencephalography (MEG) along with modern computational approaches and show how this provides new insights into the nature of ERP components and the temporal dynamics of language as a sensory and cognitive process. The full complexity of naturalistic language use cannot be captured by carefully controlled designs alone.

## Introduction

In real-life situations, the human brain is routinely confronted with complex, continuous, and multimodal sensory input. Such natural stimulation differs strikingly from traditional laboratory settings, in which test subjects are presented with controlled, impoverished, and often isolated stimuli (e.g., individual pictures or words) and often perform artificial tasks. Accordingly, cognitive neuroscience has seen an increasing trend toward more naturalistic experimental paradigms (Hasson and Honey, 2012), in which complex, dynamic stimuli (e.g., movies, natural stories) are presented without an explicit task (Hasson et al., 2004, 2008; Skipper et al., 2009; Whitney et al., 2009; Lerner et al., 2011; Brennan et al., 2012; Conroy et al., 2013; Hanke et al., 2014).

Despite being uncontrolled, naturalistic stimuli have been shown to engender distinctive and reliable patterns of brain activity (Hasson et al., 2010). However, they also pose unique challenges with respect to data analysis (Hasson and Honey, 2012; compare also the 2014 Real-life neural processing contest, in which researchers were invited to develop novel analysis techniques for brain imaging data obtained using complex, naturalistic stimulation). To date, the discussion of these challenges has focused primarily on neuroimaging data and, in the majority of cases, on visual stimulation. Naturalistic stimuli in the auditory modality, by contrast, give rise to additional, unique problems, particularly when examined using techniques with a high temporal resolution such as electroencephalography (EEG) or magnetoencephalography (MEG). Consider the case of language processing: in contrast to typical, controlled laboratory stimuli, a natural story or dialogue contains words that vary vastly in length, a stimulus property to which the temporal resolution of EEG and MEG is particularly sensitive. The characteristic unfolding over time of auditory stimuli is already evident when evoked electrophysiological responses are compared in traditional, controlled studies—the endogenous components show increased latency and a broader temporal distribution (see Wolff et al., 2008, where the same study was conducted in the auditory and visual modalities). EEG and MEG studies with naturalistic stimuli consequently tend to use the less naturalistic visual modality (segmented, rapid-serial visual presentation; Frank et al., 2015; or natural reading combined with eye-tracking; Hutzler et al., 2007; Kretzschmar et al., 2013).

Given current data-analysis techniques, these distinctive properties of the auditory modality impose severe limitations on our ability to conduct and interpret naturalistic auditory experiments, particularly when seeking to address questions related to time course information in the range of tens, or even hundreds, of milliseconds. Here, we present a new synthesis of analysis techniques that addresses this problem using linear mixed-effects modeling (LMM). We further provide an initial demonstration of the feasibility of this approach for studying auditorily presented naturalistic stimuli using electrophysiology, i.e., that it is possible to detect event-related components even with the rapid, jittered, and often overlapping epochs of a rich stimulus.

For this initial exploratory study, we focus on the N400 event-related potential (ERP), a negative potential deflection with a centro-parietal maximum and a peak latency of ∼400 ms, but the methodology applies to other ERP components as well.

## The N400

The N400 is well suited to the purposes of the present study, since it is highly robust and possibly the most researched ERP component in language (for a recent review, see Kutas and Federmeier, 2011). Although the exact mechanism(s) that the N400 indexes are still under debate, it can be broadly described as being sensitive to manipulations of expectation and its fulfillment (cf. Kutas and Federmeier, 2000, 2011; Hagoort, 2007; Lau et al., 2008; Lotze et al., 2011). This can be seen most clearly in the sensitivity of the N400 to word frequency, cloze probability, and contextual constraint but also to manipulations of more complex linguistic cues such as animacy, word order, and morphologic case as well as the interaction of these factors (Bornkessel and Schlesewsky, 2006; Bornkessel-Schlesewsky and Schlesewsky, 2009). Importantly for the examination of naturalistic stimuli, N400 amplitude is known to vary parametrically with modulations of these cues, thus making it well suited to modeling neural activity based on continuous predictors and activity fluctuations on a trial-by-trial basis (cf. Cummings et al., 2006; Roehm et al., 2013; Sassenhagen et al., 2014; Payne et al., 2015; for isolated written words, see Hauk et al., 2006; Solomyak and Marantz, 2010; for isolated spoken words, see Ettinger et al., 2014; Lewis and Poeppel, 2014; Gwilliams and Marantz, 2015; and for written words in a story, see Brennan and Pylkkänen, 2012; Brennan and Pylkkänen, 2016).

More recently, researchers have attempted to quantify expectation using measures derived from information theory, such as surprisal. These have enjoyed some success as a parsing oracle in computational psycholinguistics (Hale, 2001; Levy, 2008; for a computational approach applied to eye-tracking data, cf. Smith and Levy, 2013) and have been shown to correlate with N400 amplitude for naturalistic stimuli (real sentences taken from an eye-tracking corpus) presented with segmented visual presentation (RSVP; Frank et al., 2015).

All of these measures and manipulations show a subtlety and a contextual component that cannot be fully realized in short, carefully controlled stimuli, i.e., the very type of stimuli most dominant in the EEG literature. In the following, we show that these features can be examined successfully in a richer, naturalistic setting, despite traditional wisdom against the highly jittered and potentially overlapping epochs inherent to such settings. Specifically, we focused on the following features, all of which have been established as modulating the N400 in the extant (single sentence) literature: word frequency (higher N400 amplitude for low versus high frequency words; cf. Kutas and Federmeier, 2011), animacy (higher N400 amplitude for inanimate versus animate nouns; Weckerly and Kutas, 1999; Philipp et al., 2008; Bourguignon et al., 2012; Muralikrishnan et al., 2015), and morphologic case and its interaction with noun phrase position (higher N400 amplitude for accusative objects occurring as the first noun phrase in a sentence; Bornkessel et al., 2003; Schlesewsky et al., 2003; Wolff et al., 2008; Hörberg et al., 2013).

## Materials and Methods

### Participants

Fifty-seven right-handed, monolingually raised, German native speakers with normal hearing, mostly students at the University of Marburg and the University of Mainz, participated in the present study after giving written informed consent. Three subjects were eliminated due to technical issues, one for psychotropic medication, and one for excessive yawning, leaving a total of 52 subjects (mean age 24.2, SD 2.55; 32 women) for the final analysis.

### Experimental stimulus and procedure

Participants listened passively to a story roughly 23 min in length while looking at a fixation star. Subjects were instructed to blink as little as possible, but that it was better to blink than to tense up from discomfort. After the auditory presentation, test subjects filled out a short comprehension questionnaire to control for attentiveness.

The story recording, a slightly modified version of the German novella *Der Kuli Klimgun* by Max Dauthendey read by a trained male native speaker of German, was previously used in an fMRI study by Whitney et al. (2009). For each word in the transcribed text, a linguistically trained native speaker of German provided an annotation for the prominence features “animacy,” “morphologic case marking” (i.e., change in word form based on function in the sentence, e.g., “he” vs “him” in English; morphologic ambiguity was not resolved even if syntactically unambiguous, e.g., “it” does not change form in English, but its role is still clear from word order), “definiteness” (i.e., whether the definite article “the” was present), and “humanness” and “position” (initial or not for nominal arguments). Tags were placed at the position that the prominence information was “new”; an automated process created a duplicate tagging where the new information was repeated for the rest of its constituent phrase (e.g., copying case marking from the determiner to the head noun). Absolute (“corpus”) frequency estimates were extracted from the Leipziger Wortschatz using the Python 3 update to libleipzig-python. Relative frequencies were calculated as the ratio of orthographic tokens to orthographic types. In both cases, the resulting coding assigns a higher logarithmic frequency class to less frequent words (i.e., follows −log frequency), resulting in a positive correlation with information-theoretic measures such as surprisal. There were a total of 1682 content words in the story (used for the frequency models) and 443 noun phrases (excluding prepositional phrases and dative arguments; used for the sentence-level feature models).

### EEG recording and preprocessing

EEG data were recorded from 27 Ag/AgCl electrodes fixed in an elastic cap (Easycap GmbH) using a BrainAmp amplifier (Brain Products GmbH). Recordings were sampled at 500 Hz, referenced to the left mastoid and re-referenced to linked mastoids offline. All signal processing was performed using EEGLAB (Delorme and Makeig, 2004) and its accessory programs and plugins. Using sine-wave fitting, the EEG data were first cleaned of line noise (Cleanline plugin), and then automatically cleaned of artifacts using a procedure based on ICA (MARA; Winkler et al., 2011). Although automatic procedures have come under some criticism for being both overly und insufficiently conservative in their selection (cf. Chaumon et al., 2015), they have the distinct advantage of being (nearly) deterministic and thus completely replicable as well as faster for large numbers of subjects, as in the present study. The majority of removed components were eye movements (blinks and saccades) as well as several with a single-electrode focus, generally lateralized. As the following analysis (see below) used electrodes exclusively on the centro-parietal midline, i.e., not lateral, the removal of these components is not problematic. The ICA decomposition was performed via Adaptive-Mixture ICA on data high-pass filtered at 1 Hz (to increase stationarity) and downsampled to 100 Hz (for computational tractability; Palmer et al., 2007) and backprojected onto the original data; no rank reduction was performed and as such 27 components were extracted. Subsequently, the original data were high-pass filtered at 0.3 Hz and 1682 segments extracted per test subject, time locked to the onset of content words (cf. “open-class words” in Payne et al., 2015; Van Petten and Kutas, 1991). This filter was chosen to remove slow signal drifts as traditional baselining makes little sense in the heterogeneous environment of naturalistic stimuli (cf. Maess et al., 2016; Frank et al., 2015, who additionally found that a heavier filter helped to remove correlation between the prestimulus and component time windows; for additional discussion on baseline correction, see Alday, 2017). All filtering was performed using EEGLAB’s pop_eegfiltnew() function.

### (Lack of traditional) ERP waveforms

**Figure 1.**
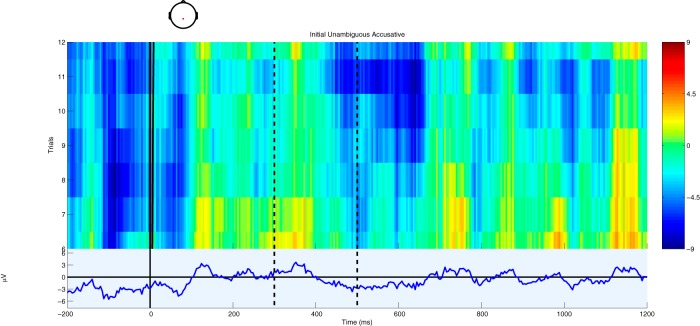
Single trial and average ERPs from electrode CPz from a single subject for unambiguous accusatives placed before a nominative. In the upper part, single trials are displayed stacked and sorted from top to bottom in decreasing orthographic length as a weak proxy for acoustic length, while the lower part displays the average ERP. Amplitude is given by color in the upper part and by the *y*-axis in the lower part. The dashed vertical lines indicate the boundaries of the N400 time window, 300 and 500 ms after stimulus onset.

In a natural story context, traditional ERP methodology with averaging and grand averaging yields waveforms that appear uninterpretable or even full of artifacts. From the perspective of continuous processing of a continuous stimulus, this is not surprising. Some information is present before word onset via context (e.g., modifiers before a noun), which leads to ERPs that seem to show an effect very close to or even before zero. Some words are longer than others, which leads to a smearing of the traditional component structure, both at a single-trial level and at the level of averages. These problems are clearly visible in Figure 1, which shows an ERP image (Jung et al., 2001) for a single participant for initial accusatives (roughly, an object-first word order), which are known to be dispreferred to initial nominatives (roughly, a subject-initial word order; Schlesewsky et al., 2003) and thus should engender an N400 effect. These eleven trials reflect the total number of trials for that particular feature constellation (initial accusative; Table 1); only with a large number of subjects and the partial pooling across conditions allowed for by mixed-effects models is it possible to examine such interactions (even then, it is difficult to achieve satisfactory power; Table 2.) Plotting additional trials from additional subjects in a single ERP image would be misleading, as this would be equivalent to a simple average across all trials, which corresponds neither to the traditional grand-average procedure nor to the mixed-model approach presented here.

**Table 1.** “Design” matrix for the sentence processing cues

**Animacy**	**Morphology**	**Position**	**Count**
inanimate	accusative	noninitial	89
inanimate	accusative	initial	4
inanimate	nominative	noninitial	8
inanimate	nominative	initial	13
inanimate	ambiguous	noninitial	99
inanimate	ambiguous	initial	52
animate	accusative	noninitial	22
animate	accusative	initial	7
animate	nominative	noninitial	8
animate	nominative	initial	16
animate	ambiguous	noninitial	39
animate	ambiguous	initial	86

Count represents the number of “trials.” The extreme lack of balance reflects natural language statistics and can only be appropriately modeled by methods using variance pooling, such as LMMs.

**Table 2.** Power calculations were performed via simulation with 1000 iterations via the simr package (Green and MacLeod, 2016**)**

Model	Predictor	Data Structure	Type of test	Lower	Upper
a	chan[cz]	Asymptotically normal	Wald *z* test	0.000	0.004
a	chan[pz]	Asymptotically normal	Wald *z* test	0.966	0.985
a	index	Asymptotically normal	Wald *z* test	0.854	0.896
a	freq.class	Asymptotically normal	Wald *z* test	0.000	0.004
a	index:freq.class	Asymptotically normal	Wald *z* test	0.000	0.004
b	chan[cz]	Asymptotically normal	Wald *z* test	0.000	0.004
b	chan[pz]	Asymptotically normal	Wald *z* test	0.966	0.985
b	index	Asymptotically normal	Wald *z* test	0.231	0.286
b	rel.freq.class	Asymptotically normal	Wald *z* test	0.000	0.004
b	index:rel.freq.class	Asymptotically normal	Wald *z* test	0.000	0.007
c	chan[cz]	Asymptotically normal	Wald *z* test	0.002	0.012
c	chan[pz]	Asymptotically normal	Wald *z* test	0.663	0.721
c	animacy[−]	Asymptotically normal	Wald *z* test	0.009	0.026
c	morphology[−]	Asymptotically normal	Wald *z* test	0.991	0.999
c	morphology[+]	Asymptotically normal	Wald *z* test	0.000	0.004
c	position[−]	Asymptotically normal	Wald *z* test	0.000	0.004
c	animacy[−]:morphology[−]	Asymptotically normal	Wald *z* test	0.010	0.027
c	animacy[−]:morphology[+]	Asymptotically normal	Wald *z* test	0.086	0.125
c	animacy[−]:position[−]	Asymptotically normal	Wald *z* test	0.000	0.004
c	morphology[−]:position[−]	Asymptotically normal	Wald *z* test	0.147	0.195
c	morphology[+]:position[−]	Asymptotically normal	Wald *z* test	0.000	0.004
c	animacy[−]:morphology[−]:position[−]	Asymptotically normal	Wald *z* test	0.006	0.020
c	animacy[−]:morphology[+]:position[−]	Asymptotically normal	Wald *z* test	0.000	0.006
d	chan	Asymptotically normal	Type-II Wald χ^2^	0.610	0.671
d	animacy	Asymptotically normal	Type-II Wald *χ* ^2^	0.179	0.230
d	morphology	Asymptotically normal	Type-II Wald *χ* ^2^	0.996	1.000
d	position	Asymptotically normal	Type-II Wald *χ* ^2^	0.964	0.985
d	animacy:morphology	Asymptotically normal	Type-II Wald *χ* ^2^	0.173	0.223
d	animacy:position	Asymptotically normal	Type-II Wald *χ* ^2^	0.182	0.233
d	morphology:position	Asymptotically normal	Type-II Wald *χ* ^2^	0.917	0.949
d	animacy:morphology:position	Asymptotically normal	Type-II Wald *χ* ^2^	0.178	0.229
e	chan	Asymptotically normal	Type-II Wald *χ* ^2^	0.611	0.672
e	index	Asymptotically normal	Type-II Wald *χ* ^2^	0.594	0.655
e	freq.class	Asymptotically normal	Type-II Wald *χ* ^2^	0.991	0.999
e	animacy	Asymptotically normal	Type-II Wald *χ* ^2^	0.037	0.065
e	morphology	Asymptotically normal	Type-II Wald *χ* ^2^	0.994	1.000
e	position	Asymptotically normal	Type-II Wald *χ* ^2^	0.922	0.953
e	index:freq.class	Asymptotically normal	Type-II Wald *χ* ^2^	0.888	0.925
e	index:animacy	Asymptotically normal	Type-II Wald *χ* ^2^	0.178	0.228
e	freq.class:animacy	Asymptotically normal	Type-II Wald *χ* ^2^	0.064	0.099
e	index:morphology	Asymptotically normal	Type-II Wald *χ* ^2^	0.461	0.523
e	freq.class:morphology	Asymptotically normal	Type-II Wald *χ* ^2^	0.980	0.994
e	animacy:morphology	Asymptotically normal	Type-II Wald *χ* ^2^	0.169	0.219
e	index:position	Asymptotically normal	Type-II Wald *χ* ^2^	0.264	0.321
e	freq.class:position	Asymptotically normal	Type-II Wald *χ* ^2^	0.025	0.049
e	animacy:position	Asymptotically normal	Type-II Wald *χ* ^2^	0.071	0.107
e	morphology:position	Asymptotically normal	Type-II Wald *χ* ^2^	0.718	0.773
e	index:freq.class:animacy	Asymptotically normal	Type-II Wald *χ* ^2^	0.037	0.065
e	index:freq.class:morphology	Asymptotically normal	Type-II Wald *χ* ^2^	0.358	0.419
e	index:animacy:morphology	Asymptotically normal	Type-II Wald *χ* ^2^	0.884	0.922
e	freq.class:animacy:morphology	Asymptotically normal	Type-II Wald *χ* ^2^	0.753	0.805
e	index:freq.class:position	Asymptotically normal	Type-II Wald *χ* ^2^	0.386	0.448
e	index:animacy:position	Asymptotically normal	Type-II Wald *χ* ^2^	0.763	0.815
e	freq.class:animacy:position	Asymptotically normal	Type-II Wald *χ* ^2^	0.345	0.406
e	index:morphology:position	Asymptotically normal	Type-II Wald *χ* ^2^	0.269	0.326
e	freq.class:morphology:position	Asymptotically normal	Type-II Wald *χ* ^2^	0.146	0.194
e	animacy:morphology:position	Asymptotically normal	Type-II Wald *χ* ^2^	0.129	0.175
e	index:freq.class:animacy:morphology	Asymptotically normal	Type-II Wald *χ* ^2^	0.259	0.316
e	index:freq.class:animacy:position	Asymptotically normal	Type-II Wald *χ* ^2^	0.435	0.497
e	index:freq.class:morphology:position	Asymptotically normal	Type-II Wald *χ* ^2^	0.414	0.476
e	index:animacy:morphology:position	Asymptotically normal	Type-II Wald *χ* ^2^	0.208	0.262
e	freq.class:animacy:morphology:position	Asymptotically normal	Type-II Wald *χ* ^2^	0.932	0.961
e	index:freq.class:animacy:morphology:position	Asymptotically normal	Type-II Wald *χ* ^2^	0.488	0.550
f	chan	Asymptotically normal	Type-II Wald *χ* ^2^	0.611	0.672
f	rel.freq.class	Asymptotically normal	Type-II Wald *χ* ^2^	0.846	0.889
f	freq.class	Asymptotically normal	Type-II Wald *χ* ^2^	0.891	0.927
f	animacy	Asymptotically normal	Type-II Wald *χ* ^2^	0.192	0.244
f	morphology	Asymptotically normal	Type-II Wald *χ* ^2^	0.996	1.000
f	position	Asymptotically normal	Type-II Wald *χ* ^2^	0.677	0.734
f	rel.freq.class:freq.class	Asymptotically normal	Type-II Wald *χ* ^2^	0.837	0.881
f	rel.freq.class:animacy	Asymptotically normal	Type-II Wald *χ* ^2^	0.994	1.000
f	freq.class:animacy	Asymptotically normal	Type-II Wald *χ* ^2^	0.334	0.395
f	rel.freq.class:morphology	Asymptotically normal	Type-II Wald *χ* ^2^	0.084	0.122
f	freq.class:morphology	Asymptotically normal	Type-II Wald *χ* ^2^	0.983	0.996
f	animacy:morphology	Asymptotically normal	Type-II Wald *χ* ^2^	0.666	0.724
f	rel.freq.class:position	Asymptotically normal	Type-II Wald *χ* ^2^	0.905	0.939
f	freq.class:position	Asymptotically normal	Type-II Wald *χ* ^2^	0.225	0.280
f	animacy:position	Asymptotically normal	Type-II Wald *χ* ^2^	0.406	0.468
f	morphology:position	Asymptotically normal	Type-II Wald *χ* ^2^	0.978	0.993
f	rel.freq.class:freq.class:animacy	Asymptotically normal	Type-II Wald *χ* ^2^	0.067	0.102
f	rel.freq.class:freq.class:morphology	Asymptotically normal	Type-II Wald *χ* ^2^	0.276	0.334
f	rel.freq.class:animacy:morphology	Asymptotically normal	Type-II Wald *χ* ^2^	0.242	0.298
f	freq.class:animacy:morphology	Asymptotically normal	Type-II Wald *χ* ^2^	0.895	0.931
f	rel.freq.class:freq.class:position	Asymptotically normal	Type-II Wald *χ* ^2^	0.361	0.422
f	rel.freq.class:animacy:position	Asymptotically normal	Type-II Wald *χ* ^2^	0.085	0.124
f	freq.class:animacy:position	Asymptotically normal	Type-II Wald *χ* ^2^	0.696	0.752
f	rel.freq.class:morphology:position	Asymptotically normal	Type-II Wald *χ* ^2^	0.061	0.095
f	freq.class:morphology:position	Asymptotically normal	Type-II Wald *χ* ^2^	0.405	0.467
f	animacy:morphology:position	Asymptotically normal	Type-II Wald *χ* ^2^	0.195	0.247
f	rel.freq.class:freq.class:animacy:morphology	Asymptotically normal	Type-II Wald *χ* ^2^	0.281	0.340
f	rel.freq.class:freq.class:animacy:position	Asymptotically normal	Type-II Wald *χ* ^2^	0.054	0.087
f	rel.freq.class:freq.class:morphology:position	Asymptotically normal	Type-II Wald *χ* ^2^	0.066	0.101
f	rel.freq.class:animacy:morphology:position	Asymptotically normal	Type-II Wald *χ* ^2^	0.336	0.397

“Lower” and “upper” are the bounds of the 95% confidence interval on the power estimates. No power estimates are provided for model comparisons, because it is not entirely clear which model to use as the simulation basis, especially for non-nested models. We note moreover that observed power calculations are problematic (Hoenig and Heisey, 2001) and indeed closely follows the observed significance (as implemented here: |t| > 2 or *p* < 0.05).

**Figure 2.**
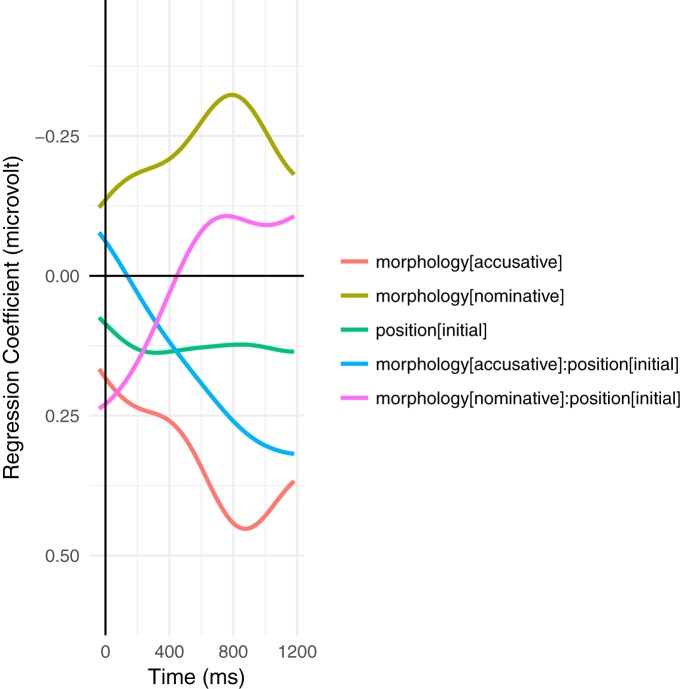
Time course of regression coefficients for the interaction between morphology and position (at the head noun of the NP), first calculated within and then averaged over participants (following the traditional grand-average methodology) with only the predictors shown for computational tractability. This is equivalent to the traditional difference wave (Smith and Kutas, 2015a). Note that already at word onset, the effects have begun to diverge; the effects at a given word in a naturalistic context reflect the sum of the context and word-local, complex interactions. Large variances in word length enhance this effect.

**Figure 3.**
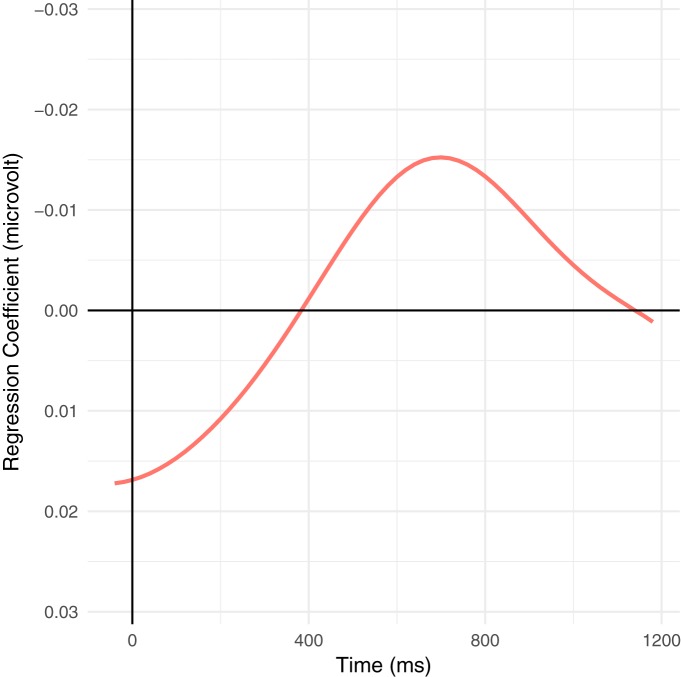
Time course of regression coefficients for the effect of frequency (logarithmic class), first calculated within and then averaged over participants (following the traditional grand-average methodology) with only the predictors shown for computational tractability. This is analogous to the traditional difference wave (Smith and Kutas, 2015a), but instead of the difference between binary classes represents the average difference between frequency classes, i.e., the average difference in the wave form for every order-of-magnitude reduction in frequency. Note that already at word onset, the effects have begun to diverge; the effects at a given word in a naturalistic context reflect the sum of the context and word-local, complex interactions. Large variances in word length enhance this effect.

**Figure 4.**
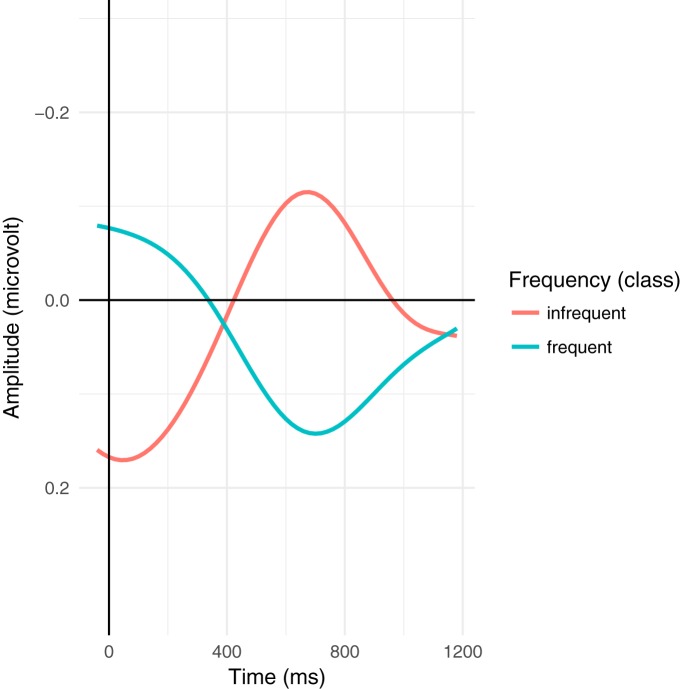
Grand average plot for the upper and lower tertiles of frequency (logarithmic class). Note that already at word onset, the effects have begun to diverge; the effects at a given word in a naturalistic context reflect the sum of the context and word-local, complex interactions. Large variances in word length enhance this effect. Nonetheless, the overall effect of frequency is so large that the change overcomes the initial offsets. This is visible as the change in sign for the regression coefficients in Figure 3.

Despite these difficulties, a modulation of the ERP signal is nonetheless detectable in the N400 time window as triangular/skewed stripes following the sorting by orthographic length. This leads to a broad, shallow negative deflection in the average wave form. Plots based on a variation of the rERP method (Smith and Kutas, 2015a), which are essentially difference waves, make this effect somewhat more apparent (Figs. 2, 3), but are somewhat misleading as they are based on simple effects (without covariates), averaged over subjects, instead of using the partial pooling of mixed-effects models to improve estimates for unbalanced designs. As such, they do not reflect the full complex interactions of the naturalistic environment as modeled below. Similarly, Figure 4 shows the ERPs for the upper and lower tertiles of frequency (thus avoiding some boundary issues present in the traditional median split). Although the ERPs start with a large initial offset, the effect of frequency is large enough to overcome this offset. This is shown in the rERP plots when the regression coefficients (the difference wave) change sign, i.e., cross zero.

### Data analysis

We examined single trial mean amplitude in the time window 300–500 ms, a typical time window for the N400 effect (Kutas and Federmeier, 2011; cf. Frank et al., 2015; Payne et al., 2015; for other single-trial analyses in traditional paradigms, see Bishop and Hardiman, 2010; Pernet et al., 2011). This time window was chosen based purely on the literature and not by examining plots from the current study to avoid any issues related to circularity (cf. Kriegeskorte et al., 2010; Vul and Pashler, 2012; Tiedt et al., 2016; Luck and Gaspelin, 2017). To simplify the analysis, both computationally and in terms of comprehensibility, only data from the electrodes Cz, CPz, and Pz were used, following the centro-parietal distribution of the N400 (cf. Payne et al., 2015; for exploratory and demonstration purposes with generalized additive mixed-effects models, see the single-electrode analysis in Tremblay and Newman, 2015). Single-trial epoch averages from these electrodes were analyzed together using LMMs (Pinheiro and Bates, 2000; Bates et al., 2015b).

### Statistical methods

Results were analyzed using LMMs. These present several advantages over traditional repeated-measures ANOVA for the exploration presented here. First, they yield quantitative results, estimating the actual difference between conditions instead of merely the significance of the difference. While it is possible to calculate effect sizes, etc. from ANOVA results, this is generally a *post hoc* test and not delivered by the ANOVA procedure directly. Moreover, mixed-effects models estimate parameters in a quantitative model framework directly, and not just effect sizes, and accommodate shrinkage and other issues related to the Stein’s paradox (Stein, 1956; Efron and Morris, 1977), which simple summary statistics like the grand mean do not do.

Second, they can easily accommodate both quantitative and qualitative independent variables, allowing us to integrate measures such as frequency without relying on dichotomization and the associated loss of power (cf. MacCallum et al., 2002). Finally, they are better able to accommodate unbalanced designs than traditional ANOVA methods.

Note that a full introduction to mixed-effect modeling is beyond the scope of this paper. A basic understanding of LMMs would thus be beneficial to the reader for the interpretation of what follows. It is, however, not essential: we presuppose only a basic familiarity with simple regression techniques. Note, in particular, that the fixed-effects coefficients in a mixed-effects model are interpreted exactly as in a classical regression model. We therefore only include explanations where mixed-effects regression differs fundamentally from classical regression. For introductions to mixed-effects modeling, we refer the interested reader to the 2008 special issue of the *Journal of Memory and Language* on “Emerging Data Analysis” (Volume 59, Number 4) for a broad introduction and to Payne et al. (2015) for EEG.

### Random-effects structure

For the analysis presented here, we use a minimal LMM with a single random-effects term for the intercept of the individual subjects. This is equivalent to assuming that all subjects react the same way to each experimental manipulation but may have different “baseline” activity. This is a plausible assumption for an initial exploration, where we focus less on interindividual variation and instead focus on the feasibility of measuring population-level effects across subjects. Furthermore, this is not in violation of Barr et al. (2013)’s advice, which is explicitly directed at confirmative studies. The reduced random-effects structure reduces the number of parameters to estimate, which (1) greatly increases the computational tractability of the exploration at hand and (2) allows us to focus the relatively low power of this experimental setup on the parameters of interest (cf. Bates et al., 2015a). (We nonetheless note that the observed power for some effects was quite high, but power suffered for higher level interactions as well as more strongly unbalanced features such as animacy; Table 2.)

We omit a random-effect term for “item” as there are no items in the traditional psycholinguistic sense here (Clark, 1973). A random effect for “lexeme” is also not appropriate because while some lexemes appear multiple times (e.g., “Ali,” the name of the title character), many lexemes appear only once and this would lead to overparameterization (i.e., modeling the present data better at the expense of being able to generalize to new data).

A single main (fixed) effect for electrode was introduced into the model. The three electrodes used are close enough together that they should all have correlated and highly similar values and so that topographical interactions should not be an issue and can thus be omitted, reducing the loss of power and increased computational complexity from additional parameters. This also accommodates variation due minor differences in physiology and cap placement between subjects better than a single-electrode analysis (cf. “optimized averaging” in Rousselet and Pernet, 2011).

### Contrast coding

Categorical variables were encoded with sum encoding (i.e., ANOVA-style coding), such that the model coefficient represents the size of the contrast from a given predictor level to the (grand) mean (represented by the intercept). For a two-level predictor, this is exactly half the difference between the two levels (because the mean is equidistant from both points).

As indicated above, the dependent measure is the single-trial average amplitude in the epoch from 300 to 500 ms after stimulus onset.

For simpler models, we present the full model summary, including an estimation of the intersubject variance and all estimated coefficients for the fixed effects, but for more complicated models, we present a contour plot of the effects as modeled (i.e., the predictions from the LMM) along with a brief selection of the strongest effects, as revealed by Type-II Wald *χ* ^2^ tests (i.e., with car::Anova(); Fox and Weisberg, 2011). Type-II Wald tests have a number of problems (cf. Fox, 2016, pages 724–725, 737–738, and discussions on R-SIG-mixed-models), but even assuming that their results yield an anticonservative estimate, we can use them to get a rough impression of the overall effect structure (cf. Bolker et al., 2009). Using *χ* ^2^ instead of *F* variant avoids issues in estimating denominator degrees of freedom in unbalanced designs, both mathematical (cf. Bates et al., 2015b) and computational, and is analogous to treating the *t* value as a *z* value for the individual coefficients (see below). Model comparisons, or, more precisely, comparisons of model fit, were performed using the Akaike Information Criterion (AIC; Akaike, 1974), the Bayesian Information Criterion (BIC; Schwarz, 1978) and log-likelihood. AIC and BIC include a penalty for additional parameters and thus provide an integrated measure of fit and parsimony. For nested models, this comparison was performed as a likelihood-ratio test, but non-nested models lack a significance test for comparing fit. We do not include pseudo *R* ^2^ values because these are problematic at best and misleading at worst in an LMM context. (The difficulty in defining an appropriate *R* ^2^ for LMM is intuitively related to the difficulties in defining correlations in a repeated measures context—should we compute correlation across subjects or within subjects and then average or something else entirely? Simpson’s paradox precludes a clear answer to this dilemma.)

For the model summaries, we view |t| > 2 (i.e., the estimate of the coefficient is more than twice as large as the error in the estimate) as being indicative of a precise estimate in the sense that the estimate is distinguishable from noise. (Note that we are using the strict technical meaning of “precise,” which does not necessarily imply “accurate.”) We view |t| < 2 as being imprecise estimates, which may be an indicator of low power or of a generally trivial effect. (We note that Baayen et al. (2008) use |t| > 2 as approximating the 5% significance level: this is equivalent to treating the *t* values as *z* values.) For the Type-II Wald tests, we use the *p* values as a rough indication of the quality of the estimate across all levels of a factor (i.e., how well the predictor can be distinguished from noise). This will become clearer with an example, and so we begin with a well-known modulator of the N400: frequency of a word in the language as a whole before turning to more complex predictors.

### Experimental “manipulations”

In the following, we examine several classic N400 effects, beginning with simple models of frequency and its relation to length of context. We show that the longer, naturalistic stimulus already allows us to view even concepts such as frequency in a more subtle fashion. Next, we examine complex interactions between sentence-level features that are rarely manipulated in more than a 2 × 2 parametric manner with minimal context in the literature and show that these interactions are important. Finally, we combine the sentence-level feature model with frequency to show that it is possible to model all these effects simultaneously, thus providing a way to statistically control for frequency effects rather than treating them as confounds (cf. Sassenhagen and Alday, 2016).

In particular, we examine the relative fits of a model based on corpus frequency versus versus a model based on relative frequency, including in both a predictor for index within the story. We then examine the effects of several higher-level cues to sentence interpretation (animacy, case marking, and word order) to determine whether our methodology is also suited to examining neural activity related to the interpretation of linguistically expressed events. Psycholinguistic studies using behavioral methods have demonstrated that such cues play an important role in determining real-time sentence interpretation (e.g., with respect to the role of a participant in the event being described; a human is a more likely event instigator, as is an entity that is mentioned early rather than late in a sentence, etc.) and, hence, expectations about upcoming parts of the stimulus (Bates et al., 1982; MacWhinney et al., 1984). Electrophysiological evidence has added support to this claim, with an increased N400 amplitude for dispreferred yet grammatically correct constructions (e.g., for accusative-initial sentences in several languages including German, Swedish, and Japanese, see Bornkessel et al., 2003; Schlesewsky et al., 2003; Wolff et al., 2008; Hörberg et al., 2013; for animacy effects in English, Chinese, and Tamil, see Weckerly and Kutas, 1999; Philipp et al., 2008; Bourguignon et al., 2012; Muralikrishnan et al., 2015). These cues are largely independent of any particular linguistic or sentence-processing theory, although they do play a central role in some theories. Observing these features in a natural story context both demonstrates that such naturalistic designs are in principle possible and allows for the first examination of complex interactions between multiple features.

While the frequency-based analyses used all 1682 content words, the analysis of sentence-level features was restricted to the 443 full noun phrases occurring as main arguments of verbs that were in the nominative or accusative case (roughly “subjects” and “objects,” not including indirect objects, e.g., the difference between “I” and “me” in English). This matches previous work most closely and avoids more difficult cases where the theory is not quite as developed (i.e., what is the role of animacy in prepositional phrases?). The resultant distribution (for each test subject) can be found in Table 1. For each of these features, we use the (sum-coded) contrast for dispreferred: inanimate, noninitial position, or unambiguous accusative configurations compared to the (grand) mean (sum coding tests main and not simple effects; see contrast coding). The particular arrangement dispreferred > (grand) mean structures the model such that the contrasts align with increased N400 activity. (The converse arrangement preferred > (grand) mean would yield a model with coefficients indexing decreased N400 activity.) For morphology, there is an additional neutral classification for ambiguous case marking, and there are thus two contrasts for the unambiguous cases: accusative (dispreferred) > (grand) mean and nominative (preferred) > (grand) mean.

## Results

### Frequency

We first examine the well-established effect of frequency on N400 amplitude (for a review, see Kutas and Federmeier, 2011), the results of which are presented in Tables 3, 4. Interestingly, both measures of frequency provided similar model fit with similar log likelihoods (and thus similar AIC and BIC as both models had the same number of parameters; Table 5).

**Table 3.** Summary of model fit for (corpus) frequency class and index (ordinal position) in the time window 300–500 ms from stimulus onset using all content words^a^

Linear mixed model fit by maximum likelihood				
AIC	BIC	logLik	Deviance	
2043327	2043410	−1021655	2043311	
Scaled residuals:				
Min	1Q	Median	3Q	Max
−24.19	−0.49	−0.01	0.49	12.54
Random effects:				
Groups	Name	Variance	SD	
subj	(Intercept)	0.04	0.19	
Residual		141.06	11.88	
Number of obs: 262392, groups: subj, 52.				
Fixed effects:				
	Estimate	SE	*t* value	
(Intercept)	0.037	0.13	0.28	
chan[cz]	−0.029	0.033	−0.89	
chan[pz]	0.13	0.033	4	
index	0.00043	0.00014	3.1	
corpus	−0.02	0.0093	−2.2	
index:corpus	−2.7e−05	9.9e−06	−2.7	

**Table 4.** Summary of model fit for relative frequency class and index (ordinal position) in the time window 300–500 ms from stimulus onset using all content words^b^

Linear mixed model fit by maximum likelihood				
AIC	BIC	logLik	Deviance	
2043374	2043457	−1021679	2043358	
Scaled residuals:				
Min	1Q	Median	3Q	Max
−24.2	−0.49	−0.01	0.49	12.55
Random effects:				
Groups	Name	Variance	SD	
subj	(Intercept)	0.04	0.19	
Residual		141.09	11.88	
Number of obs: 262392, groups: subj, 52.				
Fixed effects:				
	Estimate	SE	*t* value	
(Intercept)	0.17	0.17	0.98	
chan[cz]	−0.029	0.033	−0.89	
chan[pz]	0.13	0.033	4	
index	0.00023	0.00018	1.3	
relative	−0.068	0.028	−2.4	
index:relative	−2.5e−05	3e−05	−0.86	

**Table 5.** Comparison of models for corpus and relative frequency

	df	AIC	BIC	logLik
m.rel.index	8	2043373	2043457	−1021678
m.freq.index	8	2043326	2043410	−1021655

Both models yield similar fits as evidenced by log-likelihood, AIC, and BIC. Model names reflect the predictor used; ‘rel’ refers to relative frequency and ‘freq’ refers to corpus frequency.

### Corpus frequency

The frequency of a word in the language as whole, corpus frequency, is known to correlate with N400 amplitude and to interact with cloze probability (for a review, see Kutas and Federmeier, 2011). Using the logarithmic frequency classes from the Leipzig Wortschatz, we can see in Table 3 that corpus frequency has a small, but observable effect (only −0.02 µV per frequency class, but *t* = −2.2 in the N400 time window). This means that, for each frequency class, ERP responses diverge by a further −0.02 µV from the grand mean as represented by the intercept.

**Figure 5.**
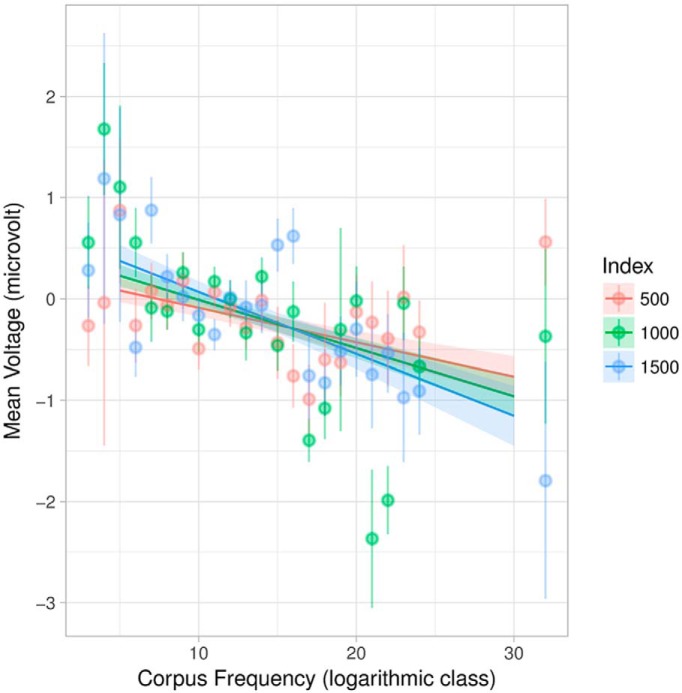
Plot of effects for corpus frequency interacting with index (ordinal position in the story). Shaded areas indicate 95% confidence intervals. Light points are grand averages by participants over all trials; the corresponding lines are standard error of the (grand) mean. Index is divided into tertiles and plotted in an overlap to show the interaction. There is an increasing negativity with decreasing frequency (higher logarithmic class), which is weakly affected by position in the story.

The negative-going interaction effect for corpus frequency and ordinal position (index) reflects the diminishing impact of frequency over the course of the story. At the sentence level, there is evidence that ordinal position modulates the role of frequency (Van Petten and Kutas, 1990; Payne et al., 2015); and this is also observable here across the entire story, albeit weakly (−0.000027µV, *t* = −2.7). This is exactly what the literature predicts: frequency is not dominant in context-rich environments but, nevertheless, plays a distinct role (cf. Dambacher et al., 2006; Kutas and Federmeier, 2011). Short stimuli presented out of context are dominated by boundary effects, e.g., the complete lack of context at the initial word and wrap-up effects at the final word, but longer naturalistic stimuli are not. This is also visible in Figure 5, in which the regression lines are closer to parallel than perpendicular.

Comparing Figures 3–5, we see that the frequency effect in Figure 5 (and thus also Table 3) appears slightly stronger than in Figures 3, 4. In Figure 5, the estimates for each participant affects the estimates for all other participants via partial pooling (“sharing” information across subjects; (this tends to pull or “shrink” the predictions for individual subjects toward the grand mean and is thus called “shrinkage”), which helps provide better estimates low information conditions, such as when there are few and/or uninformative trials (particularly relevant for rare constellations of sentence-level features below; uninformative trials arise, e.g., when little signal is left over after artifact correction). Figures 3, 5 both use continuous estimates of frequency, which avoids issues in dichotomization and thus better models ‘middle’ frequencies, which are often overlooked in studies contrasting “high” versus “low” frequency and are completely absent in Figure 4. Finally, Figure 5 uses a 200-ms windowed average for the single trial data, while Figures 3, 4 use minimal slices of time (discrete samples). The windowed average serves as a low-pass filter, eliminating high-frequency noise and, more importantly for naturalistic auditory stimulation, smoothes jitter due to variation in word length, phrase length, etc. Using a single, fixed time interval also frees up the *x*-axis for the continuous presentation of frequency. In this sense, Figure 3 reflects a “snapshot” of the frequency effect at each time point in form of the regression coefficient with time varying along the *x*-axis, while Figure 5 presents a “snapshot” at a single interval in time with frequency varying along the *x*-axis.

### Relative frequency

The relative frequency of a word in a story is also known to correlate with N400 amplitude (cf. Van Petten et al., 1991, who found a repetition priming effect for words repeated in natural reading). This is seen indirectly in repetition priming (which is essentially a minimal, binary context) and information-theoretic surprisal, which can be seen as a refinement of relative frequency.

**Figure 6.**
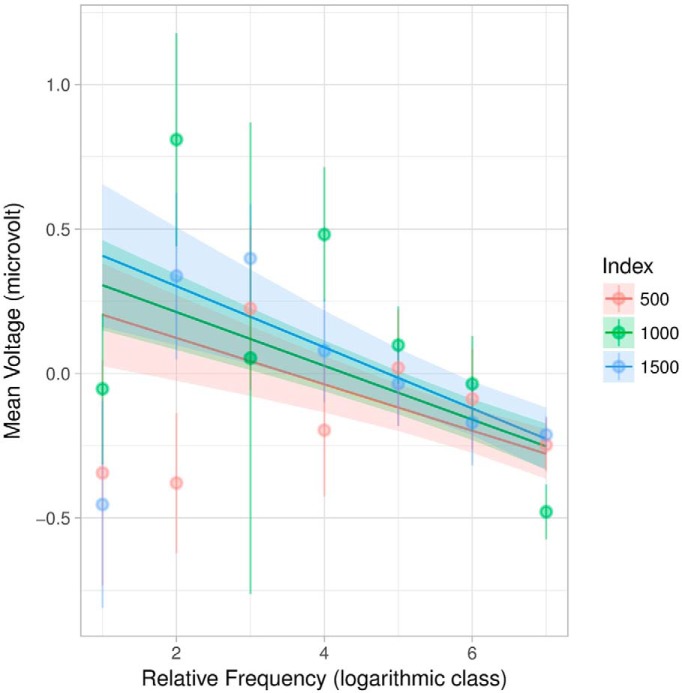
Plot of effects for relative frequency interacting with index. Shaded areas indicate 95% confidence intervals. Light points are grand averages by participants over trials; the corresponding lines are standard error of the (grand) mean. Index is divided into tertiles.

For the model presented in Table 4, relative frequency was divided into logarithmic classes using the same algorithm as for corpus frequency, but applied exclusively to the smaller “corpus” of the story. Interestingly, the overall effect sizes (coefficient estimates) are similar to those from the corpus frequency model, although the main effect for index and its interaction with frequency are less precise (larger standard error and thus |t| < 2). This interaction is visible in Figure 6 as the slow convergence of the lines at higher frequency classes, i.e., internally rarer words.

### Animacy, case marking, and word order

Examining sentence-level cues, we largely find results consistent with previous studies (Table 6; summarized with Wald tests in Table 7). From the model summary, we see main effects for both types both types of unambiguous case marking, with a negativity for unambiguous nominative/preferred (−0.35 µV, *t* = −3.1) and a positivity for unambiguous accusative/dispreferred (+0.53 µV, *t* = 4.5), which at first seems to contradict previous evidence that dispreferred cue forms elicit a negativity (for accusative-initial sentences in several languages including German, Swedish, and Japanese, see Bornkessel et al., 2003; Schlesewsky et al., 2003; Wolff et al., 2008; Hörberg et al., 2013). This somewhat surprising result is quickly explained by the interaction between morphology and position, which shows a negativity for the dispreferred late-nominative (i.e., initial-accusative) word order (−0.37 µV, *t* = −3.2). The missing main and interaction effects for animacy at first seems contrary to previous findings (for animacy effects in English, Chinese and Tamil, see Weckerly and Kutas, 1999; Philipp et al., 2008; Bourguignon et al., 2012; Muralikrishnan et al., 2015), but not surprising given the limited data and the number of interactions modeled here, which allows for the effect to be divided among several coefficients. This may also result from imbalance in the emergent “design” in a naturalistic stimulus.

**Table 6.** Summary of model fit for linguistic cues (animacy, morphology, linear position) known to elicit N400-like effects^c^

Linear mixed model fit by maximum likelihood				
AIC	BIC	logLik	Deviance	
538127	538273	−269047	538095	
Scaled residuals:				
Min	1Q	Median	3Q	Max
−18.56	−0.5	−0.01	0.49	10.65
Random effects:				
Groups	Name	Variance	SD	
subj	(Intercept)	0.15	0.39	
Residual		140.86	11.87	
Number of obs: 69108, groups: subj, 52.				
Fixed effects:				
	Estimate	SE	*t* value	
(Intercept)	−0.15	0.093	−1.6	
chan[cz]	−0.05	0.064	−0.78	
chan[pz]	0.16	0.064	2.5	
animacy[−]	−0.0068	0.075	−0.091	
morphology[−]	0.53	0.12	4.5	
morphology[+]	−0.35	0.11	−3.1	
position[−]	−0.36	0.075	−4.8	
animacy[−]:morphology[−]	−0.026	0.12	−0.22	
animacy[−]:morphology[+]	0.084	0.11	0.74	
animacy[−]:position[−]	−0.13	0.075	−1.7	
morphology[−]:position[−]	0.12	0.12	0.99	
morphology[+]:position[−]	−0.37	0.11	−3.2	
animacy[−]:morphology[−]:position[−]	−0.022	0.12	−0.19	
animacy[−]:morphology[+]:position[−]	−0.091	0.11	−0.8	

Dependent variable are single-trial means in the time window 300–500 ms from stimulus onset using only subjects and (direct) objects. For animacy and position, the coefficients are named for the dispreferred condition (note the minus sign) and represent the contrast dispreferred > mean.” Morphology also has an additional “neutral” level for ambiguous case marking, and so the coefficients represent the contrast from the respective marked conditions (note the minus and plus signs for dispreferred/unambiguous accusative and preferred/unambiguous nominative) to the (grand) mean.

**Table 7.** Type-II Wald tests for the model presented in Table 6^d^

	*χ*^2^	df	Pr(>*χ*^2^)
chan	6.66	2	0.0357
animacy	1.34	1	0.248
morphology	31.48	2	<0.001
position	15.17	1	<0.001
animacy:morphology	1.75	2	0.416
animacy:position	1.23	1	0.267
morphology:position	14.62	2	<0.001
animacy:morphology:position	2.00	2	0.368

The Wald tests show similar results in a more succinct fashion but do not indicate directionality or size of the effect (*p* values are not measures of effect size) nor the constituent components of an interaction. For brevity, results from more complex models are presented only with these Type-II Wald tests.

### Covariates, not confounds: complementing linguistic features with distributional properties

We extend the model for interacting sentence features with other distributional covariates, such as frequency and index. Not only does this allow for statistical control of potential confounds inherent to a naturalistic stimulus, it also allows us to consider the subtle interactions present in language outside of the laboratory setting. Crucially, it also provides a first step in addressing the driving force behind “inherently confounded” effects in traditional laboratory studies. At a syntactic level, this includes questions such as whether certain feature constellations are dispreferred in themselves or because of their lower occurrence. At a lexical level, this includes questions such as whether effects for animacy are simply the result of the overall higher (corpus) frequency of animate nouns.

### Index and corpus frequency

**Figure 7.**
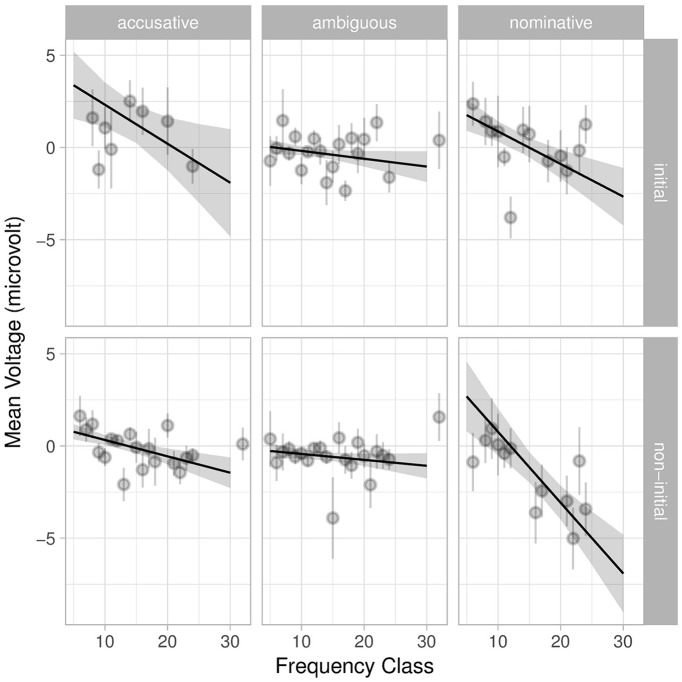
Interaction of position, morphology, and corpus frequency from the full sentence-feature model with index and frequency class. Shaded areas indicate 95% confidence intervals. Light gray points are grand averages by participants over all trials; the corresponding lines are standard error of the (grand) mean. Interactions with position show themselves as differences between the top and bottom rows, while interactions with morphology show themselves as differences between columns.

**Figure 8.**
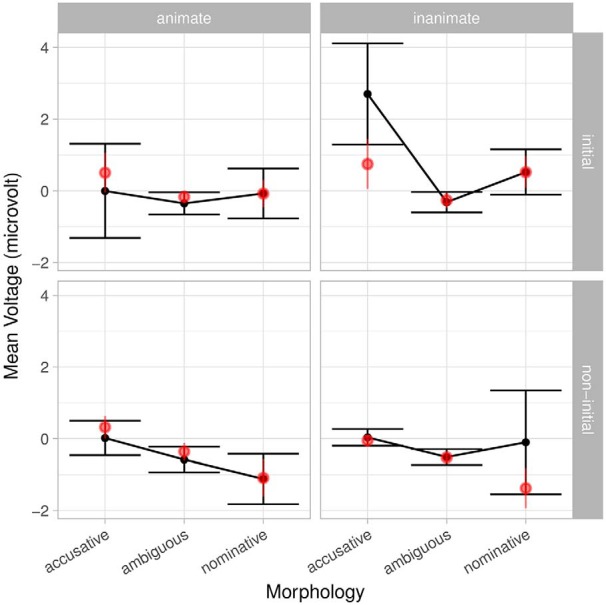
Interaction of animacy, morphology and position from the full sentence-feature model with index and frequency class. Bars indicate 95% confidence intervals. Light red points are grand averages by participants over all trials; the corresponding lines are standard error of the (grand) mean. Interactions with position show themselves as differences between the top and bottom rows, while interactions with animacy show themselves as differences between columns.

Including the covariates index and corpus frequency improves the model fit (Table 8). Figures 7–8 show selected effects from this model; selected Wald tests can be found in Table 9.

**Table 8.** Model comparison for linguistic-cue based models, extended with (1) index and corpus frequency or (2) corpus and relative frequency

	df	AIC	BIC	logLik	Deviance	χ^2^	χ^2^ df	Pr(>χ^2^)
prom	16	538126	538273	−269047	538094			
prom.rel.freq	50	538042	538499	−268971	537942	152.68	34	<0.001
prom.freq.index	52	538034	538509	−268965	537930	11.77	2	0.00278

Note that the basic model is nested within both of the larger models, but the larger models are not nested and so the results of the likelihood-ratio test must be carefully interpreted. Model names reflect the predictor used; ‘rel’ refers to relative frequency and ‘freq’ refers to corpus frequency, while ‘prom’ indicates ‘prominence’, i.e. linguistic cues.

**Table 9.** Type-II Wald tests for the clearest effects in the model combining index, (corpus) frequency, and linguistic cues^e^

	χ^2^	df	Pr(>χ^2^)
chan	6.68	2	0.0355
index	4.94	1	0.0262
corpus	20.47	1	<0.001
morphology	28.25	2	<0.001
position	11.98	1	<0.001
index:corpus	10.68	1	0.00108
corpus:morphology	19.64	2	<0.001
morphology:position	8.85	2	0.012
index:animacy:morphology	13.21	2	0.00135
corpus:animacy:morphology	9.13	2	0.0104
index:animacy:position	8.02	1	0.00462
corpus:animacy:morphology:position	14.81	2	<0.001

In this model, we find main effects for index, corpus frequency, morphology and position. There is no main effect for animacy; however, there are several interactions involving animacy. Interestingly, there is a three-way interaction between corpus frequency, animacy and morphology (as well as a four-way interaction with position), which highlight the combined effects of animacy and frequency, despite their inherent confounding (characters in natural stories tend to be animate) and the correlation between animacy and frequency (in this story, Kendall’s *τ* = −0.24). The interaction between morphology and position is again present (Figure 7). Morphology also interacts with frequency individually and in the aforementioned four-way interaction with animacy and position (Figure 8). We avoid interpreting these interactions further but note that they are compatible with results in the literature and suggest that a complete account of language cannot be reduced to either frequency or morphosyntax.

### Word length

Because of convergence issues, it was not possible to create a maximum model including orthographic length, index, corpus frequency, and all the linguistic cues, but the model with corpus frequency and orthographic length as covariates for the prominence features shows a similar set of effects. This again serves as a validity check that the effects for the linguistic cues are not merely the result of confounds with other properties of the stimulus.

### Corpus and relative frequency

We can also examine the interplay between linguistic cues and the two types of frequency in a single model, which had a better fit to the data than the more basic model, but a slightly worse fit than the model with index and corpus frequency (Table 8). Due to convergence issues, it was not possible to include index or orthographic length in this model, but nonetheless several interesting patterns emerge (for Wald tests, see Table 10).

**Table 10.** Type-II Wald tests for the clearest effects in the model combining linguistic cues with both corpus and relative frequency^f^

	χ^2^	df	Pr(>χ^2^)
chan	6.68	2	0.0355
relative	9.46	1	0.0021
corpus	11.49	1	<0.001
morphology	34.44	2	<0.001
position	6.20	1	0.0128
relative:corpus	9.65	1	0.00189
relative:animacy	24.73	1	<0.001
corpus:morphology	20.13	2	<0.001
animacy:morphology	7.44	2	0.0242
relative:position	10.88	1	<0.001
morphology:position	21.47	2	<0.001
corpus:animacy:morphology	12.40	2	0.00203
corpus:animacy:position	6.77	1	0.00926
corpus:animacy:morphology:position	11.96	2	0.00253

There are main effects for both types of frequency as well as morphology and position; additionally, corpus and relative frequency interact with each other. The interaction between morphology and position is again present as well as several interactions with animacy and a four-way interaction between all three features and corpus frequency.

## Discussion

### The present approach: examining complex influences within a fixed epoch

It is somewhat surprising that it is possible to extract effects in such a heterogeneous and noisy environment. Part of the problem with the type of presentation in Figure 1 is that the influences on N400 (and, more generally, ERP) amplitude are many, including frequency, and this three-dimensional representation (time on the *x*-axis, trial number sorted by orthographic length on the *y*-axis, and amplitude as color, or equivalently, on the *z*-axis) shows only some of them. Some hint of this complexity is visible in the trends between trials – the limited coherence of vertical stripes across trials reflects the sorting according to orthographic length. Unsorted, the stripes are greatly diminished. Similarly, other patterns emerge when we (simultaneously) sort by other variables, but our ability to represent more dimensions graphically is restricted.

A further complication is the inclusion of continuous predictors. Traditional graphical displays, and statistical techniques, are best suited for categorical predictors, which we can encode with different colors, line types or even subplots. However, the mixed-effects models are capable of incorporating many dimensions simultaneously, including continuous dimensions like frequency, which have been traditionally difficult to present as an ERP without resorting to methods like dichotomization (for a similar but complementary approach using continuous-time regression, see Smith and Kutas, 2015a,b; for a similar approach at the sentence level for a continuous-measure reanalysis of an older, dichotomously analyzed study, see Payne et al., 2015). In other words, traditional graphical representations of ERPs have difficulty displaying more complex effects and interactions.

Our approach is to pick a fixed time-window, freeing up the horizontal axis for something other than time, which fits well with the epoch-based regression approach used here and in Payne et al. (2015). Displays of the regression at a particular time point are also level curves at a particular time and provide clarity about the shape of the effect at a particular time, but are less useful for exploring the time course of the ERP. Nonetheless, this perspective allows us to study the modulation of the ERP in a given epoch via more complex influences, such as those that arise in a natural story context. The implications of this perspective, complex influences in a fixed epoch, are discussed more fully below.

### Frequency is dynamic

Somewhat surprisingly, the model for relative frequency with index provides nearly as good a fit as the model for corpus frequency (Table 5). Adopting a Bayesian perspective on the role of prior information (here: frequency), this result is less puzzling. From a Bayesian perspective, corpus frequency is a nearly universally applicable but weakly informative prior on the word, while the relative frequency is (part of) a local prior on the word. This is clearly seen in the interaction with position in the story (index). This is in line with previous sentence-level findings that frequency effects are strongest early on (cf. Payne et al., 2015). Thus, (corpus) frequency makes a small but measurable contribution in a rich context, while it tends to dominate in more restricted contexts. Relative frequency becomes a more accurate model of the world, i.e., a more informative prior, as the length of the context increases. Corpus frequency is thus in some sense an approximation of the relative frequency calculated over the context of an average speaker’s lifetime of language input.

In this sense, we can say that frequency is dynamic and not a static, inherent property of a word. In the absence of local context, frequency is calculated according to the most general context available – the sum total of language input. With increasing local context, a narrower context for calculating frequency is determined, increasingly cut down from the global language input (which now of course includes the new local context). From this perspective, it is less surprising that a model incorporating the development of relative frequency over time yields results that are nearly as good as a model based on the well-established effect of corpus frequency. Frequency is an approximation for expectation, and a larger context leads to expectation that is better predicted from that context than from general trends.

### Covariates and confounds: language is about interaction

In addition to demonstrating that this approach allows us to replicate effects that are well known from more controlled experiments, the naturalistic story environment revealed complex feature interactions that have not hitherto been reported and yet modulate these previously reported effects. Firstly, the rich story context revealed a more subtle perspective on effects of word frequency, by allowing us to contrast corpus frequency with relative frequency within the story and how this evolves as the story unfolds. Interestingly, this analysis allowed us to conclude that an increasingly specific local context provides as good a model for word expectability as a word’s global (corpus) frequency. Secondly, we observed interactions between frequency measures and the sentence-level features examined here. Specifically, as shown in Figure 6, effects of index (relative position in the story) and frequency appear to be most pronounced for arguments bearing actor features of some kind (i.e., animates and inanimate nominatives). This finding extends the results of Frenzel et al. (2015), which showed that word-level actorhood cues (e.g., a king has a higher actor potency than a beggar) interact with frequency such that lexical actorhood effects on the N400 were more pronounced with increasing frequency of occurrence. We interpret this previous finding as demonstrating that increasing familiarity with a concept, as reflected by higher corpus frequency, leads to an increasing familiarity with the actor potency of a noun. The present results indicate that a similar relation may hold for more abstract classes of actor-related features such as animacy and case.

### Implications for electrophysiological research in cognitive neuroscience: ERP components as ongoing processes

Thus far, we demonstrated that the synthesis of increasingly tractable computational techniques (mixed-effects models, automatic artifact correction with independent-component analysis) leads to a tractable approach to analyzing electrophysiological data collected in response to a naturalistic auditory stimulus (a natural story). Strikingly, the current results mirror a number of well-established findings from traditional, highly controlled studies. This is somewhat surprising given the large amount of jitter in naturalistic stimuli. The words themselves have different lengths and different phonological and acoustic features; moreover, the phrases have different lengths, which are often longer than in traditional experiments. This leads to the information carried by the acoustic-phonological signal being more broadly and unevenly distributed in time. Yet, we still see clear effects at a fixed latency, which seems to be at odds with traditional notions of ERPs as successive, if occasionally overlapping events (i.e., components), reflecting various (perhaps somewhat parallel) processing stages. (While modern ERP theories do not assume discrete events and thus easily allow for continuous modulation, the common intuition seems to be based on a weak-form of ERPology (cf. Luck, 2005) with discrete, if overlapping, components.) In the following, we discuss the implications of our results for the interpretation of ERP responses in cognitive neuroscience research, both in a naturalistic auditory environment and beyond.

From the traditional perspective, that ERPs are the sum of discrete components, individual components within the electrophysiological signal (e.g., the N200, N400, P300, and P600 to name just a small selection of examples) are interpreted as indexing particular cognitive processes which occur at certain, clearly defined times within the overall time course of processing (for a recent review in the language domain, see Friederici, 2011). However, ERP data recorded in response to naturalistic, auditory language challenge this traditional view: in contrast to ERPs in studies employing RSVP, components no longer appear as well-defined peaks during ongoing auditory stimulation. This applies equally to the early exogenous components and to endogenous components.

Let us first consider the exogenous components. The fact that these no longer appear during continuous auditory stimulation other than at stimulus onset does not mean that the neurocognitive processes indexed by these early components do not take place later in the stimulus, but rather that their form is no longer abrupt enough to be visually distinct from other signals in the EEG. The abruptness of stimulus presentation in RSVP leads to the abruptness of the components, but continuous stimulation, as in a naturalistic paradigm, leads to a continuous modulation of the ERP waveform without the typical peaks of RSVP.

More precisely, the relevant continuity is not that of the stimulus itself, but rather of the information it carries. In RSVP, all external information for a given presentation unit is immediately available, although there may be certain latencies involved in processing this information and connecting to other sources of information (e.g., binding together multimodal aspects of conceptual knowledge). Thus, as the information passes through the processing system, it is available in its entirety and there are sharp increases in neural activity corresponding to this flood of new information resulting in sharp peaks. In auditory presentation, the amount of external information is transmitted over time (instead of over space), and thus the clear peaks fall away as the incoming information percolates continuously through the processing system, yielding smaller and temporally less well-defined modulations of the ERP. In summary, we propose that the appearance of ERP components as small modulations or large peaks is a result of the relative change in the degree of information processed. In studies employing visual presentation, time-locking to recognition point (van den Brink and Hagoort, 2004; Wolff et al., 2008), or employing other similar jitter-controlling measures in auditory presentation, ERPs thus reflect the state of processing at the climax of (local) information input.

Overall, this perspective is compatible with the predictive coding framework (cf. Friston, 2005), according to which predicted stimuli lead to an attenuation of neural activity in comparison to stimuli that engender a prediction error or that were simply not predicted. In this framework, non-predicted sensory input carries a higher information content than predicted input, and this correlates with increased activity of relevant neuronal populations as well as higher ERP amplitudes.

## Conclusion

We have demonstrated the feasibility of studying the electrophysiology of speech processing with a naturalistic stimulus through a synthesis of modern computational techniques. More directly, we have demonstrated that against traditional wisdom, it is possible to detect event-related components even with the rapid, jittered, and often overlapping epochs of a rich stimulus. The replication of well-known effects served as a proof of concept, while initial exploration of the more complex interactions possible in a rich context suggested new courses of study. Surprisingly, we found robust manipulations at a fixed latency from stimulus onset despite the extreme jitter from differences in word and phrase length. This suggests that ERP responses should be viewed as continuous modulations and not discrete, yet overlapping waveforms.

## Synthesis

Reviewing Editor: Lila Davachi, New York University Decisions are customarily a result of the Reviewing Editor and the peer reviewers coming together and discussing their recommendations until a consensus is reached. When revisions are invited, a fact-based synthesis statement explaining their decision and outlining what is needed to prepare a revision will be listed below. The following reviewer(s) agreed to reveal their identity: Christian Fiebach, Jonathan Brennan

This is a very interesting paper - mainly from a methods point of view, as it demonstrates indeed the feasibility of working with continuous naturalistic stimuli. Similar work has been published in the last years for the visual domain (e.g., Frank et al., 2015), however using more complex modeling of the language stimulus. Both reviewers found the statistics strong and careful but also noted that the paper does not highlight the novelty of the findings or approach. One reviewer writes: “Against this background, it may not be clear why the current study is methodologically novel. It is! But the paper does not make the strongest case. On one view, the argument is that narrative stimuli, in which dynamic predictions are paired with rapid, jittered, and often overlapping epochs, might simply be too noisy to detect event-related components. This study compellingly rules out this reasonable possibility. This sort of motivation, which is touched on around lines 347-348, could be fore-fronted.'

In addition, other related work should be cited:. In addition to the work using fMRI and naturalistic stimuli cited in the text, previous work has used single-trial analysis with electrophysiological data when subjects comprehended isolated written words (Hauk et al. 2006 NeuroImage, Solomyak & Marantz 2009 J Cog Neurosci), isolated spoken words (Lewis & Poeppel 2014 Brain Lang; Ettinger et al., 2014 Brain Lang; Gwilliams & Marantz 2015 Brain Lang), and written words in a story (Brennan & Pylkkänen 2012 Neuroimage, In Press Cognitive Science). This background should be cited.

And the theoretical contributions made more clear: “There is not a clear theoretical contribution from the paper. The principle results concern the effect of word frequency on the N400, its interaction with word position (Van Petten & Kutas, 1990 Mem Cognit), and various factor implicated in word-order preferences that have been explored by Bornkessel-Scheslewsky and colleagues. As far as I could tell, the fact that these effects are observable in a naturalistic story-listening context is consistent with standard ERP models (Luck 2005, 2014).”

From the statistical point of view, the manuscript is very convincing. On the other hand, given the rich data set available to the authors, it seems to me as an unnecessary restriction of their approach to only analyze average activity in a single time window for only three electrodes. Computing power, in my view, cannot motivate such restricted data analyses any more, as probably every university has computing clusters that can run R and similar software. So from this perspective, the reported analyses appear to be unnecessarily restricted to me. (See also below my question concerning trial numbers and power.)

Details of reviewers concerns below:

1. The authors give no explicit information about trial numbers. It can be partly reconstructed, i.e., by deriving the number of observations from the tables, taking into account that three observations existed per trial (= 3 electrodes; anything else to consider?), but this is not necessarily obvious for all readers. Given that the authors seem to have always at least 150 trials per analysis (is this correct?) - why do you yourself describe this paradigm as statistically underpowered? In general, it would be really helpful to state such fundamental information more explicitly.

2. As already mentioned above, the figures are quite un-intuitive. I do understand that figures like Fig. 4-7 represent the LMM effects in realistic ways. However, the authors in my view should also make efforts to find ways to make these results accessible to the 'Joe Doe cognitive neuroscientist'.

In addition to the comments above, I did have a concern with how the study is framed and the types of conclusions that are highlighted. Most of this critique is rhetorical, and could be addressed in a re-write. In brief: the bulk of the results focuses on “sanity check” findings like the frequency effects. The more interesting findings, espcially those related to sentence-processing, are not framed in a way that makes their import easy to grasp.

(1) There is extensive discussion of the word-frequency effect (estimated in different ways) and its interaction with position in the story. This is presented as a “proof of concept” but takes up the bulk of the results section, with little theoretical upshot. The observation that “frequency is dynamic” and that listeners are sensitive to local frequencies and to repetition effect is, of course, well known. It is consistent with standard information theoretic views of sentence processing where frequency is recast as the unigram backoff of a richer prior over upcoming linguistic input. In sum: this is a reasonable point, but it is not a novel contribution.

The discussion of sentence-level affects may offer richer insight. But, the discussion was highly exploratory and difficult to follow. I think an exploratory approach is appropriate in this domain. But, the theoretical value of the selected features should be made clear. Why probe these features? How might these features interact in a naturalistic setting? What predictions for these interactions can be formulated? Presumably the authors are drawing on theories such as that developed in Bornkessel-Scheslewsky & Scheslewsky's 2009 book, so the theoretical bases is there. But without such an introduction, it was very difficult to draw out a take-away from this set of results. (Also, the brief discussion of these features on p. 14 belongs in the Introduction.)

(2) I did not follow how the present result supports a revision to the standard models of ERPs. As far as I could tell, the current results are consitent with existing models (just as with other single-trial analyses from sentences, such as Frank et al. 2015 Brain Lang).

(3) The structure of the paper was difficult to follow. Key ideas were not adequately discussed in the introduction (such as the sentence-level features). Significant portions of the Results section, especially concerning incremental refinements of the statistical model, should be reorganized and perhaps moved to the Methods section. One concrete possibility is to focus the Results section on the the final model, leaving discussion of different ways of measuring frequency and various motivations for exploring different models to the Methods or Introduction. Additional insights from the model-exploration can be moved to a clearly marked sub-section of the Results.

Minor:

Additional minor comments

p. 6 - “they yield quantitative results...” I didn't understand this point. ANOVAs are equally as quantitative, and can also be used to estimate effect-sizes of course (just like beta-weights).

p. 7 - How were non-categorical variables entered into the model? E.g. were frequency counts centered?

Fig 1 - I did not understand the point of this figure. If the aim is to show that the N400 is detectable in the natural story, then (a) the reasoning for selecting these twelve epochs should be offered and (b) it would be much clearer if there were a comparison showing a bigger/smaller N400 (e.g. comparing this waveform with canonical NOM-ACC occurances). Also, why not order trials by acoustic length?

p. 9 - How does the effect size for corpus frequency (0.6 muV/frequency bin) compare to the literature?

Fig 2, 3 - It may be a help to plot the actual data on these plots in addition to the model-summarizing regression lines

p. 12 - Isn't an interaction between relative frequency and index exactly what would be predicted if this effect reflects repetition priming?

p. 15 - What is the data “limited” for the analyses of sentence-level features (ln. 277)? Please include the number of datapoints for each cell in the analysis, or perhaps the average number of datapoints per participant per cell.

Fig 4,5 - These interaction plots might work better if color were used to distinguish one dimension (e.g. position) - facilitating comparison

p. 22 - “In contrast to ERPs in studies employing segmented visual presentation (RSVP), components no longer appear as well-defined peaks during ongoing auditory stimulation” This theoretical issue is not addressed by the data presented. A single-time-window analysis is not suited to tease apart whether the absence of well-defined peaks reflects decreased SNR, or a qualitative difference in how event-related activity is manifested

p. 23 - The discussion of incrementally unfolding auditory information should be grounded in the large existing literature on ERPs to speech, where clear peaks are often evident (e.g. Van Petten et al. 1999 JEP:LMC)

p. 24 - This section appears rather speculative and does not appear to be closely tied to the data presented in the study. I did not see any part of the results that tested whether information is passed continuously or discretely during incremental processing. The data appear compatible with a broad range of views.

I have just one question concerning the treatment of electrodes. P. 7 indicates that data from three separate electrodes were included in the model. The description is not quite clear. Was there a fixed effect for electrode in the model? If not, then for each <subject,word> there were three separate N400 averages, one from each electrode. These three datapoints are not independent, I am concerned that this triplicate data may artificially shrink the error terms in the model. For instance, if 10 subjects listened to 100 words under this approach, the coefficient standard errors error terms would be calculated based on 3000 datapoints rather than 1000. But this would not be correct: given the high correlation between electrodes, the true rank of the data would be significantly closer to 1000. I think it would be appropriate to average across the electrodes.

Sentence or word examples of some of the critical contrasts would be very helpful. As is, the manuscript is rather abstract
